# Altered cortical thickness associated with psychotic symptoms and cognitive profiles in involuntarily hospitalized, first-episode, drug-naive patients with schizophrenia

**DOI:** 10.3389/fpsyt.2025.1596991

**Published:** 2025-06-24

**Authors:** Ruru Tang, Wei Zhang, Xinyu Fang, Xuran Shen, Bin Zuo, Longyan Ni, Wei Yan, Rongrong Zhang, Shiping Xie

**Affiliations:** ^1^ The Affiliated Nanjing Brain Hospital, Nanjing Medical University, Nanjing, China; ^2^ Department of Psychiatry, The Second People’s Hospital of Jiangning District, Nanjing, China

**Keywords:** schizophrenia, involuntary hospitalization, drug-naive, first-episode, cognitive, cortical thickness, psychotic symptoms

## Abstract

**Objective:**

This study aims to explore the cognitive impairment characteristics, psychotic symptoms, and the relationship with alterations in cortical thickness in involuntarily hospitalized, first-episode, drug-naive schizophrenia patients.

**Methods:**

A total of 59 involuntarily hospitalized patients and 60 healthy controls were included. The MATRICS Consensus Cognitive Battery (MCCB) scale was used to evaluate cognitive function in involuntarily hospitalized patients and healthy controls. The Positive and Negative Syndrome Scale (PANSS) was utilized to evaluate psychotic symptoms in involuntarily hospitalized patients. Structural MRI scans were obtained from all participants, and the Desikan-Killiany template was used in FreeSurfer software to extract the cortical thickness values.

**Results:**

Involuntarily hospitalized patients exhibited cognitive impairments across seven cognitive domains compared to healthy controls. Additionally, these patients exhibited increased cortical thickness in the right temporal pole, left posterior cingulate gyrus, and left temporal pole compared to controls. Partial correlation analysis revealed that in the involuntarily hospitalized patients, the left posterior cingulate gyrus had a negative correlation with general symptoms, while the right temporal pole showed a positive correlation with negative symptoms. No correlation was found between cortical thickness and cognitive function in patients with involuntary hospitalization. In contrast, within the healthy control group, both the left and right temporal poles exhibited positive correlations with reasoning and problem-solving abilities.

**Conclusions:**

Our study reveals significant cognitive impairments and cortical thickness alterations in first-episode, drug-naive schizophrenia patients during their initial involuntary hospitalization. These cortical thickness alterations were significantly associated with psychotic symptoms, but not cognitive impairment. These findings suggest that cognitive dysfunction and symptom presentation in early-stage schizophrenia patients with involuntary hospitalization may be influenced by distinct neuroanatomical mechanisms.

## 1 Introduction

Schizophrenia is a chronic mental disorder with high disability and early onset (1), with a global lifetime prevalence of approximately 1% (2). It has long been a focal point and significant challenge in psychiatric research. The disorder encompasses a wide range of symptoms, including positive symptoms, negative symptoms, cognitive changes, affective symptoms, and aggressive hostility (3). Cognitive impairment is a core disabling feature of schizophrenia, with substantial deficits in attention, learning ability, memory, executive function, and social cognition (4). significantly impacting prognosis and the recovery of social function, and posing a substantial social and economic burden, especially in countries with large populations like China (5). At the same time, the severity of clinical symptoms, including hallucinations, delusions, and negative symptoms, is also directly correlated with patient prognosis and rehabilitation outcomes (6). Moreover, individuals with schizophrenia are more likely to experience involuntary hospitalization compared to those with other mental disorders (7).

Involuntary hospitalization for mental disorders has been a contentious issue since the 19th century, often sparking debates on human rights and prompting various reforms. To protect the rights of patients with mental disorders and reduce the misuse of involuntary hospitalization, China enacted its Mental Health Law in 2013 (8), which clearly defined the criteria for involuntary hospitalization. However, despite this legislative effort and evidence from studies indicating that the rate of involuntary hospitalization in psychiatric facilities has decreased after the law’s implementation (9), involuntary hospitalization continues to be the predominant form of admission, particularly for individuals with schizophrenia (7, 9). This further underscores the potential characteristics that may define this subgroup of individuals, emphasizing the need to consider this heterogeneity in scientific research.

Previous studies on involuntary hospitalization have predominantly focused on socio-demographic and clinical characteristics, community management, system improvement, disease diagnosis, readmission and so on (10–13). These studies have identified correlations between involuntary hospitalization and various patient factors, including age, gender, education, occupation, marital status, economic status, and medication compliance (14, 15). Additionally, research has shown that clinical manifestations, such as disease diagnosis, lack of self-awareness, suicidal or aggressive behavior, and irritability, are also associated with the occurrence of involuntary hospitalization (16). These findings suggest that patients undergoing involuntary hospitalization may represent subtypes with distinct biological mechanisms. Critically, prior neuroimaging studies in schizophrenia have predominantly examined mixed cohorts comprising both voluntarily and involuntarily hospitalized patients. Considering the numerous differences between voluntarily and involuntarily hospitalized schizophrenia patients, to further reduce heterogeneity, our study focused exclusively on involuntarily hospitalized patients. This approach allows us to isolate neurobiological features unique to this clinically distinct subgroup and avoid obscuring the distinct neuroanatomical mechanisms that may underlie acute behavioral dysregulation specifically observed in involuntarily hospitalized individuals. Considering the significant impact of cognitive impairment and clinical symptoms on the necessity of involuntary hospitalization and treatment outcomes, cortical thickness, as a critical marker of neural structure, is closely associated with cognitive function and the severity of clinical symptoms. However, limited research has explored the correlation between cortical thickness and both cognitive characteristics and clinical symptoms.

The relationship between cortical thickness and cognitive functioning has been extensively studied in various populations. Studies have demonstrated positive correlations between cortical thickness in the left paratriangular region and left insula with reasoning/problem-solving abilities in healthy populations (17). Another investigation revealed that greater cortical thickness in the prefrontal cortex is associated with enhanced executive functions, including decision-making and cognitive flexibility (18). Similarly, increased cortical thickness in the temporal lobe has been linked to improved memory performance and linguistic abilities (19). These collective findings suggest that optimal cortical thickness patterns in healthy individuals may facilitate efficient neural processing and superior cognitive performance across multiple functional domains. The relationship between cortical thickness and cognitive functioning has been observed to demonstrate increased complexity in individuals at clinical high-risk for psychosis or during early disease stages (20–22). A meta-analysis revealed that the thinning of the prefrontal and temporal cortices in individuals at clinical high risk is significantly associated with early deficits in attention and processing speed (23). However, the research by Shen et al. revealed that in the early stages of schizophrenia, no significant correlation was observed between cortical thickness and cognitive performance (24). It is worth noting that some research has also reported partial thickening of the cortex in certain brain regions at an early stage (24, 25), which may reflect compensatory neuroplasticity and could potentially help alleviate cognitive impairment to a certain extent. The majority of studies indicate that patients with chronic schizophrenia typically exhibit a decreasing trend in cortical thickness, which is often associated with more severe cognitive impairments (26–29). For instance, the volumetric reduction in the prefrontal lobe, temporal lobe, and hippocampus among chronic patients correlates with disorders in cognitive and emotional regulation (30–32). A large-scale investigation conducted by the ENIGMA consortium revealed that prefrontal thinning is consistently linked to negative symptoms and a decline in overall cognitive scores across different cultures (33). These findings underscore the heterogeneity of schizophrenia and suggest that the relationship between cortical thickness and cognitive function may vary depending on disease stage, specific brain regions involved, and the presence of additional pathological factors. To date, only a limited number of studies have indirectly explored the brain structural characteristics of involuntarily hospitalized schizophrenia patients. As involuntary hospitalized patients often exhibit self-injury, suicide or aggression. Bianca et al. demonstrated that patients with schizophrenia who engaged in self-harming behavior had significantly reduced cortical thickness in the right superior temporal gyrus, middle temporal gyrus, temporal pole, and insular cortex (34). Recent structural magnetic resonance imaging (sMRI) studies have confirmed significant changes in multiple brain regions, particularly the frontal and temporal lobes, in schizophrenia patients with aggressive behavior (35–37). One study of cortical morphology showed that schizophrenia patients with a history of violence had reduced cortical thickness in the precentral, parietal, temporal, and fusiform cortices (38). Another study suggests that schizophrenics with aggressive behavior may have specific defects in the circuitry of the prefrontal limbic system (39). The correlation between structural abnormalities observed in individuals with a history of schizophrenia and the severity of psychopathological symptoms has been established. The ENIGMA consortium study demonstrated that the thinning of the prefrontal cortex is significantly associated with negative symptoms, with the dorsolateral and medial prefrontal regions playing crucial roles in mediating these functional deficits (40). Another structural MRI study demonstrated that a thinner frontotemporal cortex was associated with elevated scores on the clinical assessment scale, independent of antipsychotic effects (41). In summary, these findings suggest that the brain structures associated with cognitive dysfunction and cortical thickness in schizophrenia patients overlap with the abnormal brain regions linked to involuntary hospitalization, highlighting the need for further research in this area.

Therefore, the present study focused on the initial involuntary hospitalization of medication-naive schizophrenia patients as the research subject, which holds significant clinical and scientific importance. On one hand, elucidating the neuropathological mechanisms underlying involuntary hospitalization in schizophrenia patients can clarify the intrinsic relationship between structural changes in specific brain regions and cognitive functions as well as clinical symptoms. On the other hand, by studying first-episode, untreated patients, we can avoid confounding factors such as long-term medication effects on brain structure and function. This approach allows for a more direct reflection of early neurobiological changes in the disease, providing a robust theoretical foundation for identifying early biomarkers and developing targeted intervention strategies, ultimately enhancing the functional prognosis of this vulnerable population.

## 2 Materials and methods

### 2.1 Subjects

A total of 59 first-episode drug-naïve schizophrenia patients were recruited from the Affiliated Brain Hospital of Nanjing Medical University between April 2017 and April 2021. The inclusion criteria for the patients were as follows: 1) A confirmed diagnosis of schizophrenia by two experienced senior psychiatrists according to the Diagnostic and Statistical Manual of Mental Disorders (DSM-5) criteria; 2) Aged between 16 and 45 years, Han ethnicity, and right-handedness; 3) First onset of psychosis within the past 24 months (17), with no prior exposure to antipsychotic medications or physical therapy such as transcranial magnetic stimulation (TMS) or electroconvulsive therapy (ECT); 4) At least 8 years of education and an IQ of 70 or above. Exclusion criteria included current pregnancy or breastfeeding, other significant medical or neurological conditions, substance abuse (alcohol or drugs), and any contraindications for MRI scanning.

Involuntarily hospitalized patients were determined in accordance with China’s Mental Health Law (2013) (8), requiring: (1) imminent risk of harm (documented evidence of self-harm or harm to others prior to admission), and (2) impaired decision-making capacity (lack of insight into illness and inability to comprehend treatment necessity, confirmed through clinical evaluations by two independent senior psychiatrists).

Sixty healthy controls were recruited through advertising in the local community. The inclusion criteria for healthy controls were as follows: 1) No personal or family history of mental illness or other genetic disorders; 2) Han nationality and right-handed; 3) Aged between 16 and 45 years. Exclusion criteria were the same as those for the patient group.

This study was approved by the Medical Research Ethics Committee of the Brain Hospital Affiliated to Nanjing Medical University. All participants provided written informed consent.

### 2.2 Clinical measurement

Demographic information, including gender, age, years of education, and detailed information about the patient’s illness (such as the age of onset, family history) was obtained by interviewing patients and supplemented by their caregivers. We used the Positive and Negative Symptom Scales (PANSS) to evaluate the psychopathological symptoms. In accordance with previous literature and guidelines from the European Psychiatric Association, this study employed a five-factor model, including the positive factor (items P1, P3, P5, G9), the negative factor (items N1, N2, N3, N4, N6), the cognitive factor (items P2, N5, G11), the excited factor (items P4, P7, G8, G14), and the depressed factor (items G2, G3, and G6) (42, 43). The cognitive function was assessed using the MATRICS Consensus Cognitive Battery (MCCB) scale (44), which included seven cognitive domains: processing speed, attention/vigilance, working memory, visual learning, verbal learning, reasoning/problem solving, and social cognition. T-scores in the seven cognitive areas were adjusted for age, gender, and years of education. The Chinese version of the Wechsler Adult Intelligence Scale (WAIS) was used to measure the IQ (45), including general knowledge, similarity, picture completion test, and block design. All clinician-rated scales were administered by trained raters. Repeated assessment of these scales maintained an interrater correlation coefficient greater than 0.8.

### 2.3 MRI data pre-processing

A 3.0 Tesla(Siemens, Skyra, Germany) magnetic resonance imaging (MRI) system was employed. During image acquisition, participants were instructed to remain awake with their eyes closed. Their heads were immobilized, and they maintained a supine position in a resting state. Throughout the scanning process, participants did not perform any specific cognitive tasks. The subsequent parameters pertain to the MRI settings utilized in the three-dimensional T1-weighted gradient echo sequence (fast field echo):repetition time (TR) = 2500 ms, echo time (TE) = 2.96 ms, flip angle (FA) = 9°, field of view (FOV) = 256 × 256 mm, slices per slab = 192, voxel size = 1.0 × 1.0 × 1.0 mm, slice thickness = 1.0 mm (17). The data preprocessing pipeline primarily comprises several key steps: noise reduction and header correction, intensity standardization, skull stripping, tissue segmentation, and registration to a standardized space. Utilizing FreeSurfer software (46, 47) to quantify cortical thickness, the essential procedures are as follows: 1) A cortical surface model is constructed based on the boundary between white matter and gray matter. 2) Topological errors in the surface, such as holes or intersections, are corrected. 3) Brain regions are segmented according to the Desikan-Killiany atlas. 4) The shortest distance from each vertex on the white matter surface to the corresponding point on the pial surface is measured. Finally, a rigorous quality control process is conducted to evaluate the reconstruction results, ensuring precise alignment of the gray/white matter boundary and identifying and excluding outliers that may arise from motion artifacts or registration errors.

### 2.4 Data analysis

Demographic and clinical characteristics were compared between groups using Student’s t-test or the chi-squared test, as appropriate. Analysis of covariance (ANCOVA) was conducted to examine the differences in all cognitive domains between the two groups, with age, gender, education level, and IQ included as covariates. Bonferroni correction was used for multiple comparisons (*p_bonf_*). ANCOVA was used to explore differences in left and right hemisphere cortical thickness between involuntarily hospitalized patients and healthy controls, with age, gender, and education level included as covariates. Partial correlation analyses were performed to investigate the associations between cortical thickness, cognitive function, and clinical features within the involuntary group. Statistical significance was set at a P value of less than 0.05. All statistical analyses were performed using SPSS version 26.0.

## 3 Results

### 3.1 Demographic and clinical measures

**Table 1** presents the demographic and clinical data of all participants. The results showed no significant differences in age, gender, education level between the two groups (all *p* > 0.05). However, the involuntarily hospitalized patients exhibited significantly lower IQ compared to healthy controls (*p* < 0.001). Regarding cognitive function, the involuntarily hospitalized patients demonstrated significantly poorer performance across all cognitive domains of the MCCB compared to the healthy controls (all *p_bonf_* < 0.001).

**Table 1:** Demographic and clinical characteristics between IHP and HCs groups.

	IHP(N=59)	HCs(N=60)	*t*/*x^2^* ^/^ *F*	*p*-value
Age (years)a	25.85 ± 7.32	26.85 ± 8.43	*t*=-0.692	*p*=0.490
Gender (M/F)^b^	51/8	51/9	*x^2^ =* 0.050	*p*=0.822
Education level^a^	13.61 ± 2.94	13.55 ± 2.84	*t*=0.114	*p*=0.910
WAIS(IQ)^a^	103.81 ± 11.43	116.70 ± 9.48	*t*=-6.699	*p*=0.000*
Handedness (R/L)	59/0	60/0		
DUP (months)	13.37 ± 8.42	NA		
Family history (yes/no)	11/48	NA		
MCCB				
Speed of processing^c^	35.15 ± 9.76	54.05 ± 8.99	*F*=55.187	*p*=0.000*
Attention/vigilance^c^	36.14 ± 11.27	48.70 ± 7.89	*F*=19.232	*p*=0.000*
Working memory^c^	34.42 ± 9.24	45.02 ± 7.69	*F*=24.840	*p*=0.000*
Visual learning^c^	40.41 ± 12.02	52.67 ± 8.24	*F*=12.610	*p*=0.001
Verbal learning^c^	35.22 ± 10.18	48.52 ± 9.65	*F*=12.254	*p*=0.001
Reasoning/problem solving^c^	40.97 ± 10.23	55.05 ± 7.12	*F*=35.746	*p*=0.000*
Social cognition^c^	34.29 ± 9.92	40.57 ± 10.49	*F*=5.781	*p*=0.018
Total^c^	28.36 ± 10.67	48.80 ± 8.44	*F*=60.270	*p*=0.000*
neurocognition	30.56 ± 10.49	51.00 ± 8.50	*F*=62.732	*p*=0.000*
PANSS				
Positive	24.76 ± 3.18	NA		
Negative	21.12 ± 3.37	NA		
General	46.85 ± 3.45	NA		
Total	92.46 ± 6.31	NA		
positive factors	16.20 ± 2.61	NA		
negative factors	18.56 ± 3.07	NA		
cognitive factors	8.80 ± 2.23	NA		
excited factors	11.98 ± 2.77	NA		
depression factors	6.95 ± 2.29	NA		

Data are presented as Mean ± SD or NA. IHP, involuntarily hospitalized patients; HCs, healthy controls; IQ, intelligence quotient; DUP, Duration of untreated psychosis; MCCB, MATRICS Consensus Cognitive Battery; PANSS, positive and negative syndrome scale; NA, not applicable; ^a^Two-sample t-test; ^b^Chi-square test; ^c^Analysis of covariance. Age, gender, education level and IQ were used as covariates to compare MCCB between the two groups. All *p* are Bonferroni corrected (*p < 0.001).

### 3.2 Comparisons of cortical thickness

**Figure 1:**
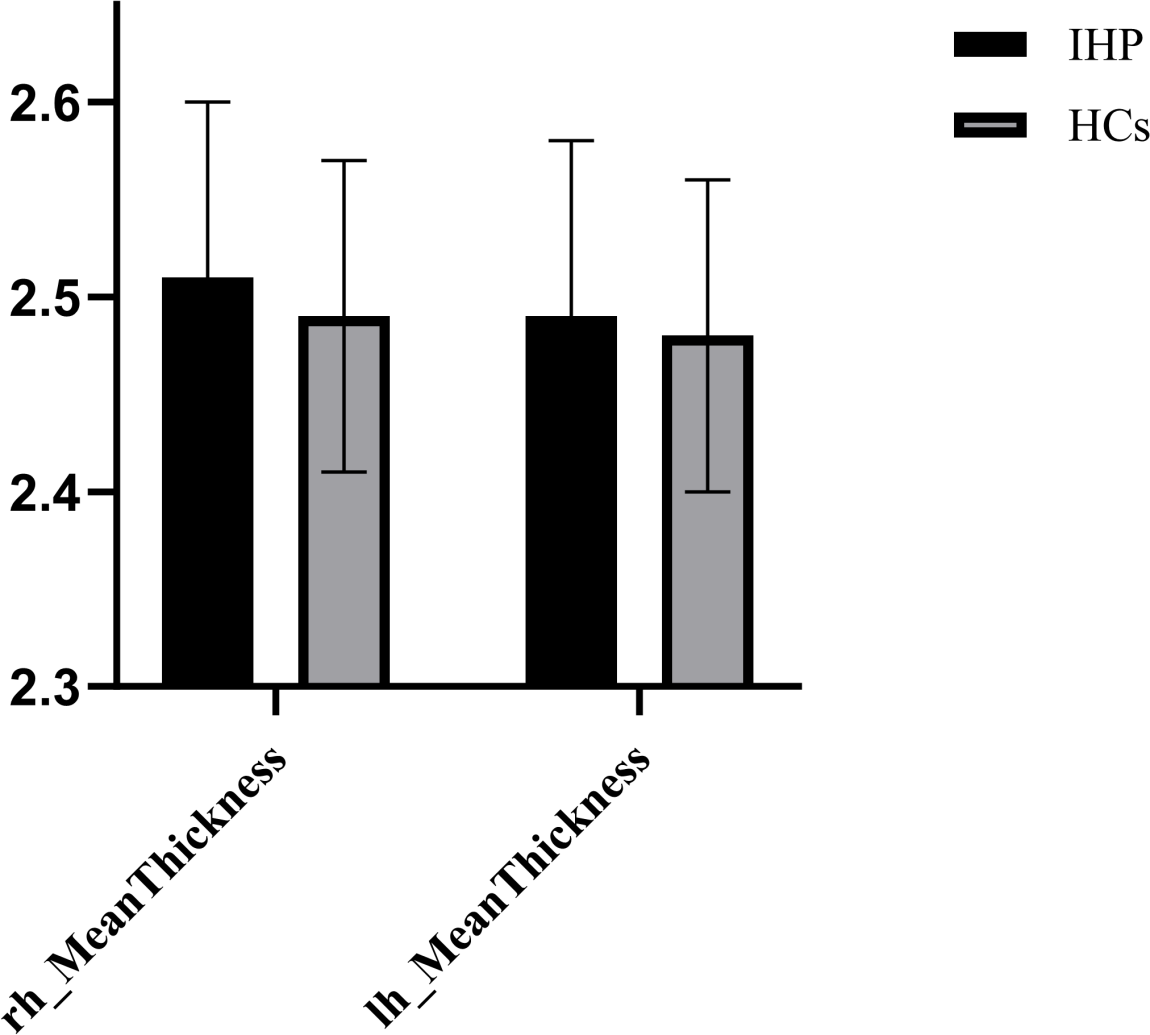
Mean cortical thickness of the left and right hemisphere. IHP, involuntarily hospitalized patients; HCs, healthy controls; lh, left hemisphere; rh, right hemisphere.

**Figure 2:**
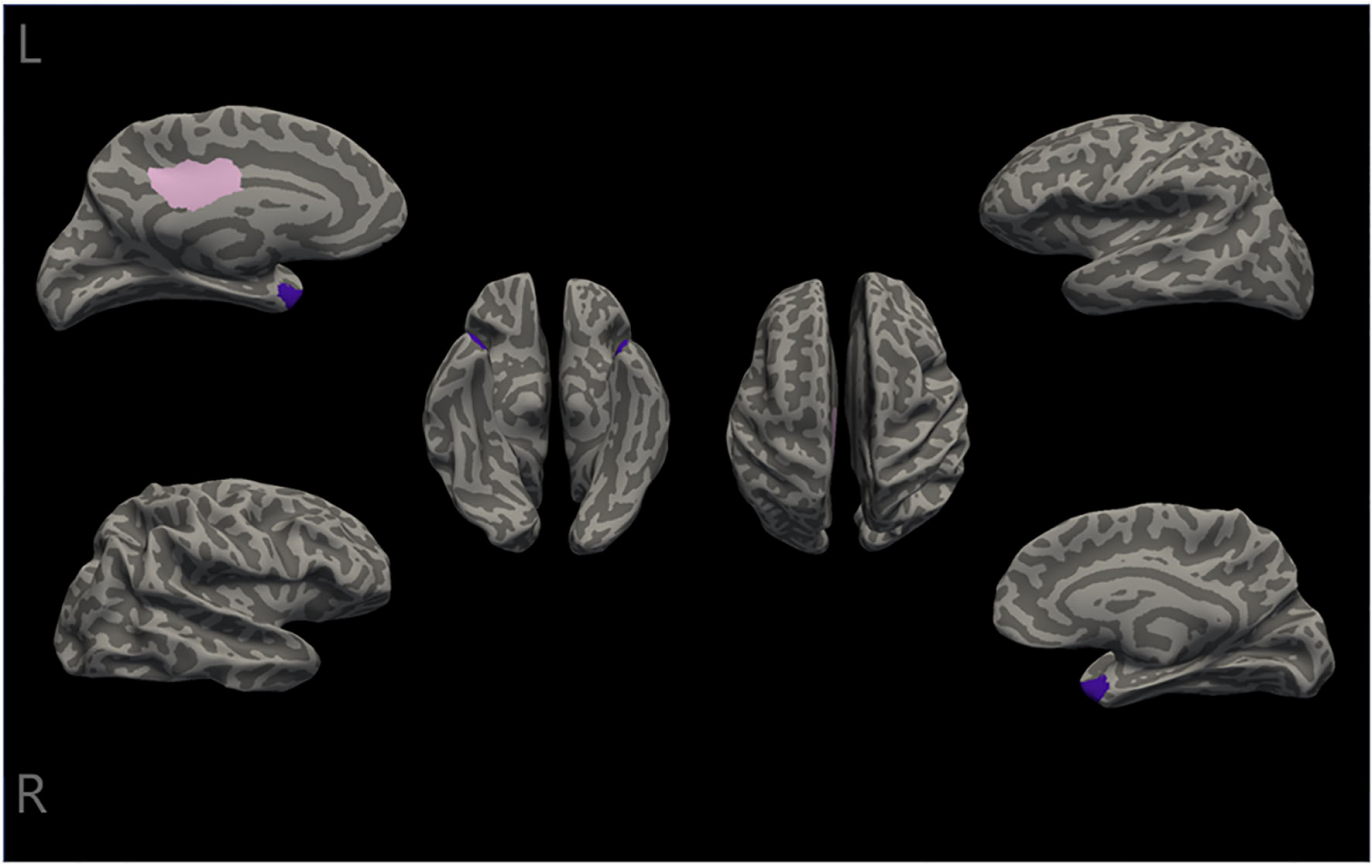
Differences in cortical thickness between the two groups. The cortical thickness of the right temporal pole, the left posterior cingulate, and the left temporal pole (the temporal pole is labeled purple, and the posterior cingulate is labeled pink) were significantly different between the two groups(all *P_bonf_* <0.05). L, left; R, Right.

In the present study, the differences in cortical thickness across 34 distinct brain structures were compared between the two groups (As shown in **Supplementary Material Table S1**). All patients underwent MRI scans within one week of admission (48). The intergroup morphometric analyses, controlling for age, gender, and education level, were performed using cortical thickness measurements. As illustrated in **Figure 1**, the mean cortical thickness was greater in the involuntary hospitalization group compared to the healthy controls for both hemispheres of the brain. Additionally, there were significant differences in the right temporal pole, the left posterior cingulate gyrus, and the left temporal pole (all *p_bonf_* <0.05) between the two groups (**Table 2**). Specifically, the involuntary hospitalization group exhibited increased cortical thickness in the right temporal pole, the left posterior cingulate gyrus, and the left temporal pole when compared to the healthy controls (**Table 2** and **Figure 2**).

**Table 2:** Cortical thickness between the two groups.

	IHP Mean ± SD	HCs Mean ± SD	*F*	*p*
rh_TP	3.75 ± 0.22	3.65 ± 0.27	5.254	0.024
lh_PC	2.49 ± 0.13	2.44 ± 0.10	4.865	0.029
lh_TP	3.65 ± 0.25	3.53 ± 0.24	7.634	0.007

rh, right hemisphere; TP, temporal pole; lh, left hemisphere; PC, posterior cingulate; IHP, involuntarily hospitalized patients; HCs, healthy controls.

### 3.3 Correlation analysis

**Figure 3:**
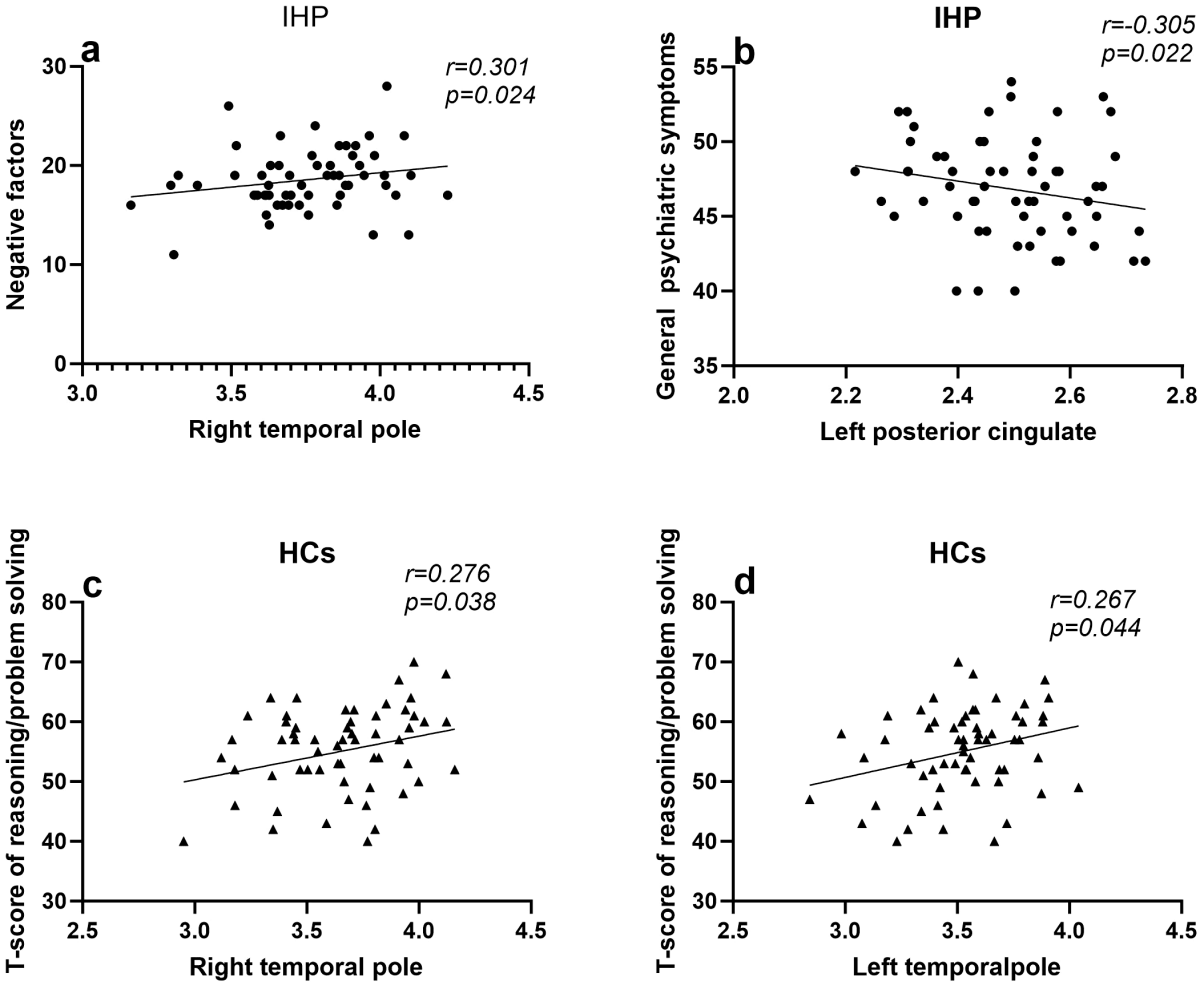
The Association between Cortical Thickness and Cognitive and Clinical Symptoms. IHP,involuntarily hospitalized patients;HCs, healthy controls; **(a)** Larger cortical thickness in the left posterior cingulate gyrus is related to a reduction in general psychiatric symptoms, as indicated by IHP (*r* = -0.305, *p* = 0.022). **(b)** Larger cortical thickness in the right temporal pole gyrus is associated with more negative factors, as indicated by IHP (*r* = 0.301, *p* = 0.024). **(c)** Larger cortical thickness in the left temporal pole gyrus is related to better reasoning/problem solving performance, as indicated by HCs (*r* = 0.267, *p* = 0.044). **(d)** Larger cortical thickness in the right temporal pole gyrus is related to better reasoning/problem solving performance, as indicated by HCs (*r* = 0.276, *p* = 0.038).

Furthermore, the partial correlation analysis was performed to investigate the relationship between aberrant cortical thickness in brain regions and clinical characteristics, including positive symptoms, negative symptoms, general psychiatric symptoms, and cognitive function. In the involuntarily hospitalized patients, the results identified a significant negative correlation between the cortical thickness of the left posterior cingulate gyrus and the severity of general psychiatric symptoms (*r* = -0.305, *p* = 0.022). Additionally, the right temporal pole showed a significant positive correlation with the severity of negative symptoms (*r* = 0.301, *p* = 0.024). However, no significant association was observed between cortical thickness and cognitive function in the involuntary hospitalization group. In contrast, within the healthy controls, cortical thickness in both the left and right temporal poles was positively correlated with reasoning/problem solving (left: *r* = 0.267, *p* = 0.044; right: *r* = 0.276, *p* = 0.038) (**Figure 3**). After Bonferroni correction was conducted, all above significant correlations did not exist.

## 4 Discussion

To the best of our knowledge, this study represents the first investigation into cognitive function alterations and cortical structural changes in first-episode, drug-naive schizophrenia patients admitted involuntarily, compared with healthy controls, and examines the relationship between regional cortical thickness changes and cognitive impairment as well as clinical symptoms in this population. The findings demonstrate that involuntarily hospitalized patients exhibit significant cognitive impairments across seven cognitive domains compared to healthy controls. Moreover, these patients showed increased cortical thickness in the right temporal pole, left posterior cingulate gyrus, and left temporal pole. Further partial correlation analysis revealed that in the involuntarily hospitalized group, cortical thickness in the left posterior cingulate gyrus was negatively correlated with general symptom severity, while the right temporal pole exhibited a positive correlation with the negative symptom factor. In contrast, within the healthy control group, cortical thickness in both the left and right temporal poles was positively correlated with reasoning and problem-solving abilities.

### 4.1 Cognitive function

As anticipated, our findings revealed significant impairments across all seven cognitive domains assessed by the MCCB, including information processing speed, attentional alertness, working memory, visual learning, verbal learning, reasoning/problem-solving, and social cognition. These results provide robust support for the “generalized cognitive deficit” hypothesis in schizophrenia (49–51). It has also been demonstrated that cognitive impairment in schizophrenia is evident independently of the influence of antipsychotic medications. The most significant impairment was observed in information processing speed, whereas the impairment in social cognition was relatively mild. The meta-analytical study conducted by Knowles et al. supports processing speed impairment as a core characteristic of cognitive deficits in individuals with schizophrenia, and underscores the significant impact of moderating variables, including medication dosage, variations in IQ, and publication year, on the effect size (52). This study recruited patients who had not yet initiated pharmacological treatment, thereby eliminating the potential confounding effects of medication. By controlling for IQ, the study compared cognitive function between the patient group and the control group. The findings further substantiate that information processing speed is a foundational component of cognitive function and has a significant impact on performance across other cognitive domains. According to a meta-analysis published in JAMA Psychiatry (51), impairment of information processing speed (Hedges’ g > 0.80) is identified as the most sensitive cognitive indicator of schizophrenia and may serve as a potential early recognition marker or outcome predictor for the disease. Abplanalp et al. showed that processing speed and facial expression recognition are the basic domains of neurocognition and social cognition respectively, suggesting that the social cognition and neurocognition of schizophrenia patients are relatively independent to a certain extent (53). Social cognition represents a complex cognitive domain involving the ability to understand and interpret others’ emotions, intentions, beliefs, and related psychological states (54, 55). Unlike neurocognition, social cognition is susceptible to a wider array of influencing factors. Although impairments in this domain may appear relatively mild, they are consistently more pronounced in affected individuals compared to healthy controls. It is important to acknowledge that the MCCB social cognitive subtest primarily evaluates emotion recognition, while higher-order theory of mind abilities, such as intention inference, are not comprehensively assessed. Some patients may preserve their capacity for recognizing basic emotions, yet they may exhibit latent deficits in more complex social scenarios.

Previous research in brain imaging has indicated that processing speed deficits may be contingent upon the efficient coordination of extensively distributed neural networks, such as the prefrontal-parietal-thalamic circuit (56–58). In this study, the observed cortical thickening in the left and right temporal poles and the left posterior cingulate gyrus in patients may reflect compensatory neural remodeling. However, the lack of concurrent increases in cortical thickness in other brain regions could result in reduced efficiency of the executive control network, thereby impairing its ability to rapidly integrate multimodal information and leading to functional deficits. In addition, social cognition depends on the interaction of the temporal pole with the amygdaloid-orbitofrontal cortex (59–62), and the thickening of bilateral temporal poles in patients in this study may partially compensate for the deficit in higher-order affective interpretation by enhancing the primary processing of social cues (such as face recognition). The overactivation of the threat perception system in involuntarily hospitalized patients who are at risk of violence may mask the objective impairment of social cognition.

### 4.2 Cortical thickness

We focused specifically on cortical thickness because the surface area and thickness of the cerebral cortex are important factors that affect the results of brain volume measurements. The surface area of the cerebral cortex is primarily influenced by genetic factors and embryonic development, exhibiting limited variation in adulthood. It is likely more closely associated with genetic predisposition to disease rather than with the progression of symptoms (63). In the majority of studies, the correlation between surface area and symptomatology in schizophrenia was found to be weak and did not exhibit statistically significant differences compared to the control group (33, 64). In contrast, cortical thickness continues to be influenced by the environment after birth (65, 66) (e.g., synaptic pruning, myelination), while key pathological processes in schizophrenia (e.g., synaptic overpruning during adolescence) may be more directly reflected in thickness changes. Therefore, the measurement of cortical thickness in patients is more sensitive.

In contrast to the majority of literature that reports cortical thinning in patients with schizophrenia (29, 67, 68), this study observed a significant increase in cortical thickness specifically in the right temporal pole, the left posterior cingulate gyrus, and the left temporal pole. These differences may reflect a distinctive characteristic of the cohort in this study: 1) Compensatory mechanisms during the early stages of the disease. Specifically, during the initial phase of the illness, the brain may respond to neural functional impairment by increasing cortical thickness as a compensatory adaptation (69). A study of cortical thickness in 128 patients with first-episode, untreated schizophrenia (70) showed that the patient group exhibited significantly lower cortical thickness, mainly in the bilateral prefrontal and parietal cortex. Meanwhile, the thickness of the bilateral anterior temporal lobe, left orbitofrontal medial cortex, and left cuneus increased. In this study, the cortical thickness was found to be both thinner and thicker, while in our study, only an increase in cortical thickness was observed, with no evidence of cortical thinning. This may be related to the specific sample group in our study. In our study, first-episode untreated schizophrenia patients who were involuntarily hospitalized exhibited violent aggression or suicidal self-injury behaviors, which may induce structural changes in specific brain regions and stronger compensatory mechanisms. 2) Specificity of Neurochemical Abnormalities: Patients who are involuntarily hospitalized may exhibit overactivation of the dopaminergic system (71) or glutamatergic toxicity, leading to localized synaptic remodeling. In animal models, acute-phase hyperglutamatergic activity has been shown to induce astrocyte proliferation and an increase in dendritic spine density (72, 73), which manifests as localized cortical thickening. Currently, the mechanisms underlying cortical thickness changes in involuntarily hospitalized patients remain unclear. Future studies should employ a range of advanced technical approaches to elucidate these mechanisms.

It is noteworthy that the clinical heterogeneity of schizophrenia underscores the need for caution when interpreting our results. While our findings highlight the importance of studying involuntary hospitalization as a distinct phenotype, they cannot fully disentangle whether the observed cortical changes are intrinsic to the disease process, secondary to acute behavioral dysregulation, or a combination of both. Future studies directly comparing involuntarily and voluntarily hospitalized patients - matched for symptom severity, illness duration, and medication status - are critical to determine whether these structural differences are unique to involuntary subgroups or reflect a continuum of disease severity.

### 4.3 Correlation

In the involuntary hospitalization group, there was a negative correlation between the thickening of the posterior cingulate gyrus and the severity of general symptoms. Prior research has demonstrated that modulating the integration of the default mode network (DMN) is associated with alleviation of symptoms (74). The posterior cingulate gyrus serves as a critical node within the DMN. Its cortical thickening may mitigate general symptoms, such as delusions, by enhancing functional coupling between the DMN and the control network (75), thereby inhibiting excessive self-referential processing. The positive correlation between the right temporal pole and negative factors suggests that overcompensation of the temporal pole may exacerbate anhedonia by inhibiting ventral striatum dopamine release (76, 77). These findings indicate a substantial correlation between alterations in cortical thickness in specific brain regions and clinical manifestations. In the involuntary hospitalization group, no significant correlation was identified between cortical thickness and cognitive characteristics. In contrast, a cross-sectional study demonstrated that cognitive impairment in patients with schizophrenia was significantly correlated with cortical thickness in five specific regions: the right medial and caudal frontal gyrus, superior frontal gyrus, fusiform gyrus, subcallosal cortex, and superior limbic gyrus (78). Previous research conducted by our group indicates a positive correlation between the thickness of the right orbitofrontal cortex and both attention/vigilance as well as visual learning ability in individuals diagnosed with schizophrenia (79). It is important to highlight that previous studies have reported a reduction in cortical thickness, whereas our study observed an increase in cortical thickness. Acute behavioral dysregulation in involuntarily hospitalized patients may trigger compensatory neuroplastic changes (69, 80), such as cortical thickening in the temporal pole or posterior cingulate gyrus, thereby achieving transient stabilization of neural circuits at the expense of functional efficiency. Such compensatory remodeling may decouple structural measures from cognitive performance, as observed in this study. This finding further implies that the clinical symptoms and cognitive impairments associated with schizophrenia may reflect distinct disease processes, each having unique relationships with cortical thickness.

Temporal pole thickness was positively correlated with reasoning/problem solving in the healthy control group. However, this association was not observed in the involuntary hospitalization group, suggesting a “structure-function decoupling” phenomenon commonly reported in schizophrenia (81). In healthy brains, there is typically a robust correlation between structural and functional connectivity (82). By contrast, in disorders such as schizophrenia, this correlation may be markedly reduced or even absent. This decoupling may be associated with diminished synaptic efficiency or neurotransmitter disturbances (83), such as insufficient GABAergic inhibition, thereby reflecting the underlying neurobiological mechanisms of cognitive dysfunction in schizophrenia.

Notably, the observed associations failed to withstand rigorous multiple comparisons correction (Bonferroni method), which may be caused by the current small sample size. Future investigations with expanded cohorts are warranted to validate these preliminary findings.

## 5 Limitation

Limitations of this study: First, the cross-sectional design precludes determining causal relationships. Second, due to the significant clinical heterogeneity observed in schizophrenia, our study was restricted to a specific clinical subgroup—involuntarily hospitalized patients. This selective inclusion limits the generalizability of the findings. Third, the lack of voluntarily hospitalized patients in our sample underscores the importance for future studies to systematically compare clinical manifestations and neuroimaging characteristics between voluntarily and involuntarily hospitalized individuals with schizophrenia. Fourth, The social cognition subtest of the MCCB may lack sufficient sensitivity to adequately assess this complex domain in individuals with schizophrenia, and accurate evaluation may require more comprehensive assessment tools. Fifthly, PANSS has notable limitations in assessing negative symptoms of schizophrenia, as it incorporates items that extend beyond the conceptual boundaries of negative symptomatology. While the scale primarily emphasizes observable behavioral manifestations, it does not comprehensively capture subjective internal experiences, thereby restricting its utility for evaluating the full spectrum of negative symptoms in clinical and research contexts. Sixthly, the relatively limited sample size may compromise the reliability and external validity of the results. Future research should incorporate multimodal neuroimaging techniques (e.g., fMRI, DTI) to explore the functional implications of structural abnormalities and monitor longitudinal alterations in cortical morphology and cognitive progression. Additionally, including voluntary hospitalization controls would facilitate distinguishing disease-specific effects from involuntary features.

## 6 Conclusions

In summary, our findings reveal that involuntarily hospitalized patients with first-episode, drug-naive schizophrenia exhibit a distinct pattern characterized by cognitive deficits, symptom dimensions, and cortical structural changes. Specifically, widespread cognitive impairments were accompanied by compensatory thickening in the temporal pole and posterior cingulate gyrus, which showed significant correlations with symptom dimensions rather than cognitive function. These results provide novel biological insights into the heterogeneity of schizophrenia and highlight the critical need for timely interventions during the acute phase to harness neuroplastic potential.

## Data Availability

The raw data supporting the conclusions of this article will be made available by the authors, without undue reservation.
