# Supplementary material for: Altered cortical thickness associated with psychotic symptoms and cognitive profiles in involuntarily hospitalized, first-episode, drug-naive patients with schizophrenia
Source: Front Psychiatry. 2025 Jun 24;16:1596991. doi: 10.3389/fpsyt.2025.1596991 (PMC12234461; doi:10.3389/fpsyt.2025.1596991)
Supplement: Supplementary file 1 [file SupplementaryFile1.docx]

Table S1 All cortical thickness between the two groups

|  | IHP  Mean ± SD | HCs  Mean±SD | *F* | *p* |
| --- | --- | --- | --- | --- |

| rh_bankssts_thickness | 2.62±0.17 | 2.59±0.15 | 0.675 | 0.413 |
| --- | --- | --- | --- | --- |
| rh_caudalanteriorcingulate_thickness | 2.41±0.17 | 2.43±0.16 | 0.629 | 0.429 |
| rh_caudalmiddlefrontal_thickness | 2.55±0.13 | 2.53±0.13 | 0.72 | 0.398 |
| rh_cuneus_thickness | 1.96±0.09 | 1.94±0.10 | 1.1 | 0.296 |
| rh_entorhinal_thickness | 3.41±0.24 | 3.42±0.31 | 0.001 | 0.981 |
| rh_fusiform_thickness | 2.78±0.14 | 2.77±0.12 | 0.321 | 0.572 |
| rh_inferiorparietal_thickness | 2.47±0.11 | 2.45±0.11 | 0.38 | 0.539 |
| rh_inferiortemporal_thickness | 2.85±0.13 | 2.84±0.10 | 0.114 | 0.736 |
| rh_isthmuscingulate_thickness | 2.25±0.15 | 2.27±0.14 | 1.054 | 0.307 |
| rh_lateraloccipital_thickness | 2.26±0.12 | 2.28±0.09 | 1.048 | 0.308 |
| rh_lateralorbitofrontal_thickness | 2.68±0.13 | 2.66±0.10 | 0.838 | 0.362 |
| rh_lingual_thickness | 2.06±0.12 | 2.04±0.11 | 1.316 | 0.254 |
| rh_medialorbitofrontal_thickness | 2.48±0.13 | 2.49±0.10 | 0.191 | 0.663 |
| rh_middletemporal_thickness | 2.88±0.13 | 2.87±0.11 | 0.132 | 0.717 |
| rh_parahippocampal_thickness | 2.66±0.19 | 2.66±0.20 | 0.006 | 0.938 |
| rh_paracentral_thickness | 2.52±0.15 | 2.48±0.14 | 2.209 | 0.14 |
| rh_parsopercularis_thickness | 2.66±0.12 | 2.65±0.12 | 0.04 | 0.842 |
| rh_parsorbitalis_thickness | 2.70±0.15 | 2.70±0.16 | 0.083 | 0.774 |
| rh_parstriangularis_thickness | 2.53±0.13 | 2.49±0.12 | 3.158 | 0.078 |
| rh_pericalcarine_thickness | 1.71±0.13 | 1.66±0.14 | 3.812 | 0.053 |
| rh_postcentral_thickness | 2.10±0.12 | 2.10±0.13 | 0.156 | 0.694 |
| rh_posteriorcingulate_thickness | 2.44±0.12 | 2.41±0.11 | 1.814 | 0.181 |
| rh_precentral_thickness | 2.56±0.15 | 2.50±0.22 | 3.65 | 0.059 |
| rh_precuneus_thickness | 2.41±0.11 | 2.39±0.11 | 0.731 | 0.394 |
| rh_rostralanteriorcingulate_thickness | 2.76±0.20 | 2.73±0.17 | 0.734 | 0.393 |
| rh_rostralmiddlefrontal_thickness | 2.38±0.10 | 2.37±0.09 | 0.11 | 0.741 |
| rh_superiorfrontal_thickness | 2.73±0.12 | 2.72±0.10 | 0.136 | 0.713 |
| rh_superiorparietal_thickness | 2.20±0.10 | 2.19±0.10 | 0.761 | 0.385 |
| rh_superiortemporal_thickness | 2.87±0.13 | 2.86±0.12 | 0.205 | 0.651 |
| rh_supramarginal_thickness | 2.54±0.12 | 2.54±0.13 | 0.168 | 0.683 |
| rh_frontalpole_thickness | 2.81±0.23 | 2.78±0.21 | 0.205 | 0.652 |
| rh_temporalpole_thickness | 3.75±0.22 | 3.65±0.27 | 5.254 | 0.024* |
| rh_transversetemporal_thickness | 2.42±0.17 | 2.41±0.22 | 0.017 | 0.898 |
| rh_insula_thickness | 3.03±0.13 | 2.99±0.12 | 2.916 | 0.09 |
| rh_MeanThickness_thickness | 2.51±0.09 | 2.49±0.08 | 0.884 | 0.349 |
| lh_bankssts_thickness | 2.49±0.14 | 2.47±0.15 | 0.332 | 0.565 |
| lh_caudalanteriorcingulate_thickness | 2.55±0.22 | 2.55±0.17 | 0.001 | 0.981 |
| lh_caudalmiddlefrontal_thickness | 2.54±0.12 | 2.53±0.12 | 0.116 | 0.734 |
| lh_cuneus_thickness | 1.90±0.12 | 1.88±0.12 | 0.879 | 0.35 |
| lh_entorhinal_thickness | 3.37±0.26 | 3.34±0.27 | 0.572 | 0.451 |
| lh_fusiform_thickness | 2.73±0.13 | 2.72±0.11 | 0.071 | 0.79 |
| lh_inferiorparietal_thickness | 2.44±0.10 | 2.44±0.10 | 0.008 | 0.929 |
| lh_inferiortemporal_thickness | 2.76±0.12 | 2.77±0.12 | 0.276 | 0.601 |
| lh_isthmuscingulate_thickness | 2.32±0.17 | 2.28±0.11 | 1.951 | 0.165 |
| lh_lateraloccipital_thickness | 2.20±0.12 | 2.21±0.10 | 0.264 | 0.609 |
| lh_lateralorbitofrontal_thickness | 2.67±0.12 | 2.69±0.12 | 1.365 | 0.245 |
| lh_lingual_thickness | 2.02±0.13 | 2.01±0.11 | 0.077 | 0.782 |
| lh_medialorbitofrontal_thickness | 2.50±0.12 | 2.50±0.10 | 0.032 | 0.859 |
| lh_middletemporal_thickness | 2.84±0.13 | 2.84±0.13 | 0.217 | 0.643 |
| lh_parahippocampal_thickness | 2.66±0.24 | 2.66±0.27 | 0.003 | 0.96 |
| lh_paracentral_thickness | 2.47±0.13 | 2.45±0.17 | 0.314 | 0.577 |
| lh_parsopercularis_thickness | 2.59±0.12 | 2.59±0.12 | 0.032 | 0.858 |
| lh_parsorbitalis_thickness | 2.64±0.18 | 2.65±0.15 | 0.412 | 0.522 |
| lh_parstriangularis_thickness | 2.46±0.13 | 2.42±0.10 | 3.728 | 0.056 |
| lh_pericalcarine_thickness | 1.69±0.15 | 1.65±0.15 | 1.545 | 0.216 |
| lh_postcentral_thickness | 2.07±0.10 | 2.10±0.11 | 1.742 | 0.19 |
| lh_posteriorcingulate_thickness | 2.49±0.13 | 2.44±0.10 | 4.865 | 0.029* |
| lh_precentral_thickness | 2.59±0.11 | 2.56±0.18 | 1.321 | 0.253 |
| lh_precuneus_thickness | 2.41±0.13 | 2.39±0.12 | 0.227 | 0.634 |
| lh_rostralanteriorcingulate_thickness | 2.75±0.16 | 2.75±0.14 | 0.008 | 0.931 |
| lh_rostralmiddlefrontal_thickness | 2.36±0.10 | 2.36±0.09 | 0.112 | 0.739 |
| lh_superiorfrontal_thickness | 2.76±0.13 | 2.75±0.10 | 0.009 | 0.924 |
| lh_superiorparietal_thickness | 2.22±0.09 | 2.21±0.10 | 0.123 | 0.727 |
| lh_superiortemporal_thickness | 2.81±0.14 | 2.80±0.13 | 0.016 | 0.899 |
| lh_supramarginal_thickness | 2.53±0.11 | 2.51±0.10 | 0.558 | 0.456 |
| lh_frontalpole_thickness | 2.80±0.20 | 2.81±0.21 | 0.065 | 0.799 |
| lh_temporalpole_thickness | 3.65±0.25 | 3.53±0.24 | 7.634 | 0.007* |
| lh_transversetemporal_thickness | 2.40±0.18 | 2.40±0.20 | 0 | 0.99 |
| lh_insula_thickness | 2.98±0.15 | 2.96±0.15 | 0.32 | 0.573 |
| lh_MeanThickness_thickness | 2.49±0.09 | 2.48±0.08 | 0.12 | 0.73 |

IHP, involuntarily hospitalized patients; HCs, healthy controls; rh, right hemisphere; lh, left hemisphere; All *p* are Bonferroni corrected (**p* < 0.05).


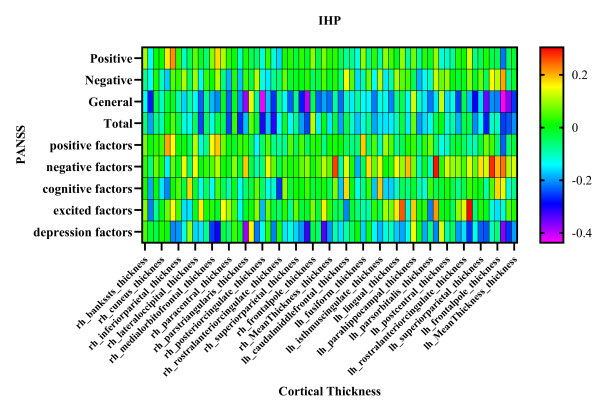

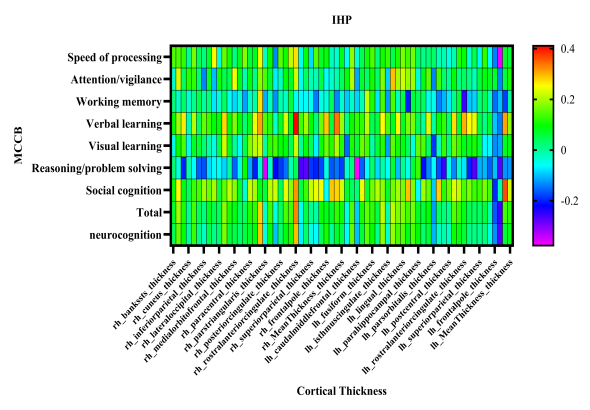


1. (b)


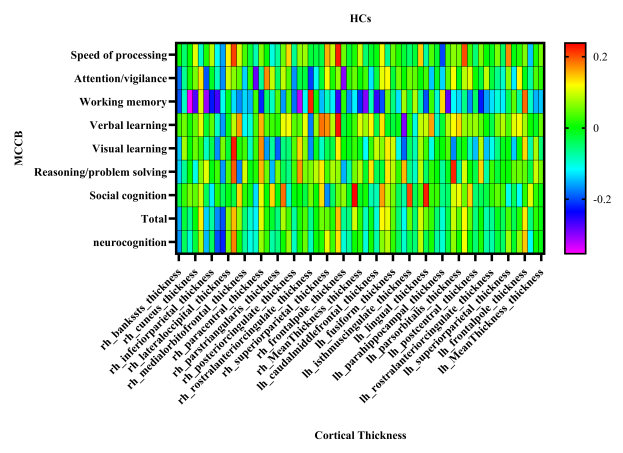


(c)

Fig. S1 The associations between the cortical thickness of brain regions without statistical significance and cognitive and clinical symptoms. IHP,involuntarily hospitalized patients;HCs, healthy controls.

Discussion of Fig.S1

We found no overlapping brain regions correlated with MCCB scores between the two groups. In healthy controls, 11 brain regions were correlated with MCCB scores, none of which were found in patients. Conversely, 30 brain regions were correlated with MCCB scores in patients, none of which existed in healthy controls. Within the patient group, reasoning/problem solving was negatively correlated with brain regions, while other MCCB subscales showed positive correlations. In healthy controls, the pattern was the reverse. Notably, brain regions positively correlated with reasoning/problem solving in healthy controls were statistically different from those in patients. These findings suggest “structure-function” alterations in brain regions of involuntarily hospitalized first-episode untreated schizophrenia patients. Moderate brain - region changes may compensate for functions, while excessive changes can cause functional disorders. We also identified 22 brain regions correlated with clinical symptoms. Negative correlations were found between general symptoms, total PANSS scores, and depressive factors with brain regions, while positive correlations existed between negative symptoms, excitatory factors, and brain regions. These correlations may reflect an early compensatory mechanism.
